# The Integrative Function of Silent Synapses on Subplate Neurons in Cortical Development and Dysfunction

**DOI:** 10.3389/fnana.2019.00041

**Published:** 2019-04-16

**Authors:** Patrick O. Kanold, Rongkang Deng, Xiangying Meng

**Affiliations:** Department of Biology, University of Maryland, College Park, MD, United States

**Keywords:** cortex, development, circuit, NMDA-receptor, silent synapse, subplate neuron

## Abstract

The thalamocortical circuit is of central importance in relaying information to the cortex. In development, subplate neurons (SPNs) form an integral part of the thalamocortical pathway. These early born cortical neurons are the first neurons to receive thalamic inputs and excite neurons in the cortical plate. This feed-forward circuit topology of SPNs supports the role of SPNs in shaping the formation and plasticity of thalamocortical connections. Recently it has been shown that SPNs also receive inputs from the developing cortical plate and project to the thalamus. The cortical inputs to SPNs in early ages are mediated by N-methyl-D-aspartate (NMDA)-receptor only containing synapses while at later ages α-amino-3-hydroxy-5-methyl-4-isoxazolepropionic acid (AMPA)-receptors are present. Thus, SPNs perform a changing integrative function over development. NMDA-receptor only synapses are crucially influenced by the resting potential and thus insults to the developing brain that causes depolarizations, e.g., hypoxia, can influence the integrative function of SPNs. Since such insults in humans cause symptoms of neurodevelopmental disorders, NMDA-receptor only synapses on SPNs might provide a crucial link between early injuries and later circuit dysfunction. We thus here review subplate associated circuits, their changing functions, and discuss possible roles in development and disease.

## Introduction

The mammalian cerebral cortex is unique in that it is a laminated structure which is thought to support hierarchical information processing. The transmission of information from the thalamus to the cortex *via* thalamocortical circuits is of central importance for information processing in the mammalian brain. In the adult, thalamic input to the cerebral cortex mainly arrives in layer 4 but inputs to other layers can exist (Meyer et al., 2010; Bruno, 2011; Sherman, 2012, 2017; Constantinople and Bruno, 2013; Ji et al., 2016; Sun et al., 2016).

Given the laminar nature of the cerebral cortex, the development of the cortical layers is a protracted process. Curiously, the development of the cortical layers occurs in an inside-out fashion such that deeper layers are generated before superficial layers. The first postmitotic neurons from the ventricular zone and the germinative zone of the rostromedial telencephalic wall migrate radially and form the so-called preplate. Subsequent rounds of cell division give rise to the excitatory neurons of the cortical plate. Since these neurons migrate towards the pia, they divide the preplate into the superficial marginal zone and the deep subplate zone (Supèr et al., 1998; Kanold and Luhmann, 2010; Pedraza et al., 2014). Even though the subplate zone is only present in the brain transiently, it plays an important role in cortical development.

**Figure 1:**
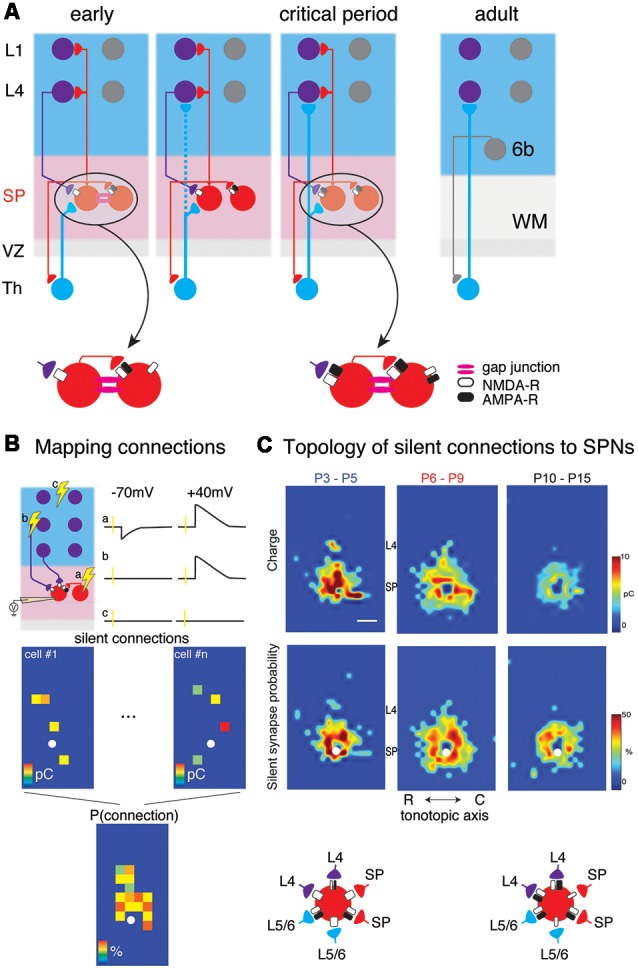
**(A)** Changing circuits between thalamus (Th), subplate (SP) and cortical layers 4 (L4) and 1 (L1). Insets show the existence of NMDA-R only “silent” synapses at early ages. **(B)** Cartoon illustration mapping of connections to subplate neurons (SPNs) *via* laser-scanning photostimulation (LSPS). Patch clamp recordings in voltage clamp are made from SPNs and locations are selectively stimulated. The SPN is recorded at −70 mV and +40 mV membrane potential at each stimulation location. Traces **(a–c)** indicate three potential outcomes of stimulation at the respective locations. **(a)** Stimulation of presynaptic locations that were connected to the SPN with AMPA and NMDARs resulted in excitatory postsynaptic currents (EPSCs) at both −70 mV and +40 mV. **(b)** Stimulation of presynaptic locations that were connected to the SPN with only NMDARs resulted in EPSCs at only +40 mV. **(c)** Stimulation of presynaptic locations that were not connected to the SPN resulted in no EPSCs at either −70 mV and +40 mV. Bottom shows calculating the spatial connection probability from maps of NMDAR-only connections (e.g., sites similar to site **b**). White circle indicates soma location of SPN. Maps of individual neurons are aligned to the soma and the probability of observing an EPSC is calculated for all relative spatial locations. **(C)** Changing topology of silent synapses over development. Shown are average connection probability and mean charge maps from LSPS experiments (from Meng et al., 2014). Cartoon below summarizes these data. At early ages, silent synapses are present between L4, L6/6, SP and SP and have a large synaptic strength. Silent synapses are most abundant at P6-P9 and at older ages, the strength of silent synapses has decreased.

Studies both in cats and rodents have shown that subplate neurons (SPNs) are responsible for the functional maturation and segregation of the thalamocortical connections (Ghosh et al., 1990; Ghosh and Shatz, 1992; McConnell et al., 1994; Kanold et al., 2003; Tolner et al., 2012), as well as for the maturation of cortical GABAergic inhibition (Kanold and Shatz, 2006), and critical period plasticity (Kanold and Shatz, 2006). This key role of SPNs is thought to stem from the fact that SPNs are the first neurons to receive thalamic inputs (Friauf and Shatz, 1991; Hanganu et al., 2002; Higashi et al., 2002, 2005; Molnár et al., 2003; Zhao et al., 2009), respond to sensory stimuli (Wess et al., 2017) and relay this information to the developing layer 4 (L4) (Kanold, 2009; Zhao et al., 2009; Butts and Kanold, 2010; Kanold and Luhmann, 2010; Deng et al., 2017; Figure 1A). Thus, the subplate might form a functional protomap of the future cortical organization (O’Leary and Borngasser, 2006; O’Leary et al., 2007; Wess et al., 2017).

Given the key position of SPNs in the thalamocortical pathway and their demonstrated role in cortical development, we here aim to review the changing circuits associated with SPNs and their possible functional relevance.

## The Subplate Zone Contains a Diverse Population of Neurons

Both glutamatergic and GABAergic neurons are found in the subplate zone. The majority of the cortex projecting SPNs are glutamatergic in ferret, while some of them are positive for GABAergic interneuron markers (Finney et al., 1998). GABAergic SPNs are also found in mouse, cat, and primate (Chun and Shatz, 1989; Antonini and Shatz, 1990; Meinecke and Rakic, 1992; Higo et al., 2007; Kanold and Luhmann, 2010). SPNs have diverse dendritic and axonal morphology. Several distinct morphological types such as bitufted and monotufted horizontal, multipolar, inverted pyramidal, polymorphous, and fusiform SPNs have been identified (Mrzljak et al., 1988; Hanganu et al., 2002; Marx et al., 2017). Some SPNs have extensive dendritic trees in the cortical plate (Friauf et al., 1990; Finney et al., 1998; Clancy and Cauller, 1999; Piñon et al., 2009; Viswanathan et al., 2012), suggesting that they are integrating synaptic information from other sources. Such sources could originate locally within the cortical plate close to the SPN soma or from distant sources, e.g., other cortical or subcortical regions. Molecular analysis has shown that SPNs express a variety of molecular markers such as CTGF, Nurr1, Cplx3, as well as others (Hoerder-Suabedissen et al., 2009, 2013; Viswanathan et al., 2012). Molecularly defined classes of SPNs have been shown to have dendritic trees in the cortical plate (Viswanathan et al., 2012) suggesting that molecular cellular identity might co-vary with morphology (reviewed in Luhmann et al., 2018).

## Outputs: Synaptic Targets of SPNs

While the subplate zone contains both glutamatergic and GABAergic neurons, ventricular zone derived SPNs are glutamatergic. SPNs send excitatory projections into the developing cortical plate and axons from SPNs target excitatory and GABAergic cells in L4 (Zhao et al., 2009; Deng et al., 2017; Figure 1A). Besides L4, SP axons are found in sub- as well as supragranular layers including layer 1 (L1) where they can potentially activate L1 neurons as well as apical dendrites of many other cortical cells (Clancy and Cauller, 1999; Viswanathan et al., 2017). Moreover, Cajal Retzius cells in L1 have been shown to receive GABAergic inputs from subplate (Myakhar et al., 2011), thus SPNs potentially target cells across the cortical column.

At young ages, SPNs are also coupled to each other as well as to some cortical plate neurons *via* gap junctions (Figure 1A; Dupont et al., 2006). In the adult cerebral cortex gap junction coupling is mostly present between GABAergic neurons (Galarreta and Hestrin, 1999, 2001, 2002; Gibson et al., 1999; Venance et al., 2000; Deans et al., 2001; Connors and Long, 2004; Butovas et al., 2006; Fukuda et al., 2006; Ma et al., 2011) while in development gap junction coupling more widespread (Niculescu and Lohmann, 2014). Gap junction coupling could synchronize neuronal activity, cause network oscillations, and contribute to circuit formation and plasticity (Peinado et al., 1993a,b; Roerig and Feller, 2000; Connors and Long, 2004; Cruikshank et al., 2005; Kotak et al., 2007; Postma et al., 2011; Niculescu and Lohmann, 2014; Yao et al., 2016). Thus, gap junctions between SPNs could synchronize local groups of SPNs and thereby amplify their effects on synaptic targets.

SPNs are also thought to pioneer the corticothalamic pathway (McConnell et al., 1994) and as such SPNs have been shown to send projections to the thalamus (Viswanathan et al., 2017; Hoerder-Suabedissen et al., 2018). The described thalamic projecting SPNs have targeted higher-order thalamic nuclei (Viswanathan et al., 2017; Hoerder-Suabedissen et al., 2018), which develop early and also support multisensory processing in early development (Henschke et al., 2018). Thus, SPNs might play a role in early multisensory integration or synchronization.

## Inputs: Subplate Neurons Integrate Cortical Information *via* a Varying Set of Synapses

Functional sources of cortical inputs to SPNs have been identified by studies in brain slices of the developing mouse auditory cortex (Viswanathan et al., 2012; Meng et al., 2014). These studies have shown that SPNs receive excitatory AMPA receptor-mediated and GABAergic inputs from the cortical plate (Viswanathan et al., 2012). In particular, these inputs originated in deep cortical layers, but subpopulations of SPNs also received inputs from L4. SPNs receiving L4 input were more prominent after thalamocortical connections to L4 were established roughly around ear opening at ~P11 in the mouse (Figure 1C).

Besides glutamatergic and GABAergic inputs, SPNs also receive neuromodulatory inputs from a variety of sources. For example, SPNs have both acetylcholine (Ach; Hanganu et al., 2002, 2009; Hanganu and Luhmann, 2004) and serotonin receptors (Kanold and Luhmann, 2010; Liao and Lee, 2011, 2014), and cholinergic as well as serotonergic fibers are present in the developing subplate (Krmpotić-Nemanić et al., 1983; Voigt and de Lima, 1991; Nakazawa et al., 1992; Mechawar and Descarries, 2001) suggesting that they form functional circuits with SPNs.

## Subplate Neurons Receive Cortical Information *via* Silent Synapses

Ionotropic glutamatergic neural transmission is the major form of excitatory neural transmission in the central nervous system. Presynaptic release of glutamate is sensed by AMPA receptors (AMPARs) and also by NMDA receptors (NMDARs) on the postsynaptic sites. Binding of glutamate alone is not enough to make NMDARs active. Membrane depolarization is also required due to Mg^2+^ block of NMDARs (Nowak et al., 1984). Thus, when glutamatergic synapses conducting currents only at depolarized membrane potential were identified, they were named as “silent synapses” (Isaac et al., 1995, 1997; Liao et al., 1995; Durand et al., 1996; Isaac, 2003). This silent synapse phenomenon is most likely due to lacking AMPARs on the postsynaptic site, but other mechanisms could exist (Kullmann and Asztely, 1998; Kullmann, 2003).

Due to the blockage by Mg^2+^ and lack of AMPARs in early development many glutamatergic synapses in several brain regions are mediated by silent synapses and the abundance of silent synapses in cells decreases over development (Isaac et al., 1997; Rumpel et al., 1998, 2004; Deng et al., 2017). Silent synapses can be turned into AMPAR-containing synapses through long-term potentiation protocols. Together, they support the hypothesis that neural activity unsilencing silent synapses is a mechanism for building mature neural circuits (Kerchner and Nicoll, 2008; Hanse et al., 2013). Indeed, the glutamatergic connections from layer 2/3 (L2/3) to L5 pyramidal neurons in the cortex are mediated by silent synapses during the first postnatal week. These NMDAR-only connections can be turned into AMPAR-containing connections by elevated network activity (Anastasiades and Butt, 2012).

**Figure 2:**
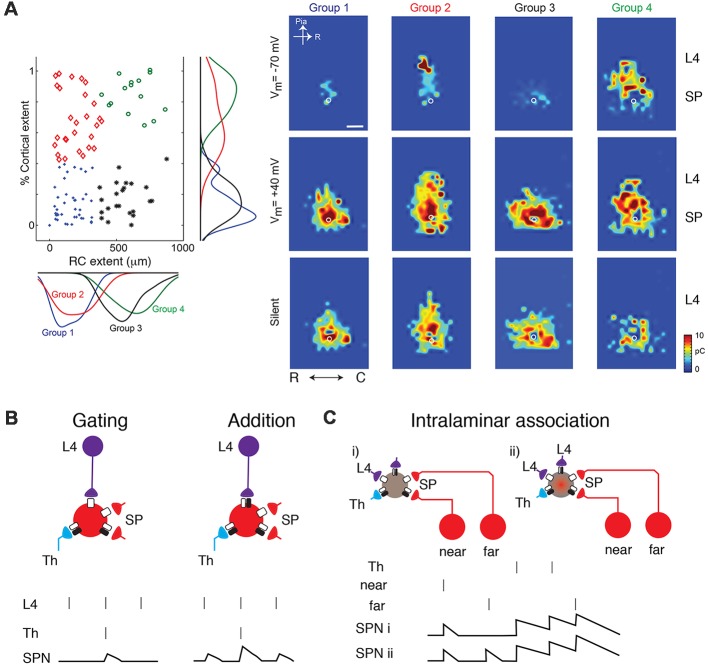
**(A)** SPNs can be classified into four groups based on their inputs from L4 (from Meng et al., 2014; no copyright permission as required for use of this image). Some classes show silent or AMPAR-containing inputs from L4 while others broadly integrate within the subplate (e.g., group 3).** (B)** Cartoon illustration how the changing presence of silent synapses on L4 inputs can change the functional association between thalamic inputs and L4 inputs. Vertical bars indicate presynaptic spikes and SPN membrane potential indicated below. At young ages, thalamic inputs might gate L4 inputs such that an SPN depolarization is only present when both thalamic and L4 activity occurs. At older aged SPN depolarizations occur when either is active and inputs sum. **(C)** Silent synapses might enable the conditional association of inputs e.g., from different locations within the same lamina.

*In vitro* studies in slices of neonatal mouse auditory cortex using Laser Scanning Photostimulation (LSPS; Figure 1B) showed that cortical input to SPNs were mediated by silent synapses for most of SPNs from postnatal day 3 (P3) to P15 (Meng et al., 2014), which encompasses the period from before the formation of thalamocortical synapses past the critical period. In these experiments, presynaptic neurons were activated by uncaging glutamate sequentially in a grid over the A1, while GABAergic transmission was blocked. By holding SPNs either at −70 mV or +40 mV the nature of the connection between the activated presynaptic neuron at each stimulus location and the recorded SPN could be probed. Presence of AMPARs results in excitatory postsynaptic currents (EPSCs) at both −70 mV and +40 mV (e.g., from within SP at stimulation site a in Figure 1B). In contrast, when only NMDARs are present, the connection is “silent” at −70 mV and EPSCs are only observed at +40 mV (stimulation site b in Figure 1B). By sequentially stimulating many locations to cover the extent of the slice, maps of NMDAR-only (“silent”) connections can be obtained. For each stimulation site, the size of the evoked EPSCs is quantified. By mapping multiple neurons, the probability of having NMDAR-only connections from each spatial location can be calculated (Figure 1B bottom). Mapping inputs to SPNs across age and calculating the spatial connection probability as well as the average strength of such a connection shows that circuits to SPNs change over development (Figure 1C). SPNs had connections that could either be NMDAR-only or AMPAR-containing. Overall, SPNs at young ages have silent connections mediating inputs from cortical cells located at larger distances from the SPN soma (Figure 1C). Many of these silent connections at early ages originated in L4. Comparing the abundance of silent connections, a transient increase in silent connections from the cortical plate occurred from P6 to P9 suggesting the formation of glutamatergic connections during this time period. A population analysis revealed that SPNs could be categorized into four different groups based on their functional connectivity patterns and in particular, their inputs from L4 (Meng et al., 2014; Figures 2A,B). Moreover, groups of SPNs varied in their amount and extent of intralaminar integration along the rostro-caudal direction, which in A1 is the dominant tonotopic axis. For example, SPNs in both groups 2 and 4 receive “silent” L4 input but vary in the amount of rostro-caudal integration and thus presumably in their functional integration across the frequency axis. These studies indicate that SPNs form a diverse integrative neural circuit and that the nature of integration varies with age. In particular, feedback inputs from L4 are mediated by NMDARs at early ages but contain AMPARs at older ages.

SPNs are not the only cerebral cortical neurons sporting silent synapses. GABAergic neurons have also been shown to have silent synapses and some of these silent synapses originate from connections from SPNs (Deng et al., 2017). Thus, the presence of silent synapses on cortical neurons seems to be a ubiquitous feature of the developing cerebral cortex.

## How Then Are Silent Connections to Spns Unsilenced?

The neural activity necessary to depolarize SPNs in order to displace the Mg^2+^ could come from depolarizing network activity during development. There are multiple non-exclusive sources of depolarizing inputs: (i) ascending sensory inputs; (ii) depolarizing GABAergic inputs; and (iii) depolarizing neuromodulatory inputs.

Ascending sensory activity mediated by AMPARs at thalamocortical terminals could provide depolarizing neural activity to SPNs and regulate the unsilencing of silent synapses on SPNs. Perturbing sensory activity resulted in the prolonged existence of silent synapses in the visual and somatosensory cortices (Ashby and Isaac, 2011; Funahashi et al., 2013; Han et al., 2017). Similarly, removing auditory inputs by cochlear ablation in early life altered the spatial distribution of AMPAR- and NMDAR-mediated connections on GABAergic neurons in the auditory cortex (Deng et al., 2017). In deaf mice, AMPAR-mediated connections were present for more distal connections from L2/3 neurons while L5/6 connections originated from more proximal locations. The spatial pattern of NMDAR-only connections was also altered in deaf animals. This suggests that glutamatergic synapse maturation is regulated with input specificity but that both extrinsic and intrinsic cortical activity such as sensory-evoked activity as well as endogenous cortical activity might play a role.

A second source of depolarizing activity might be provided by GABAergic inputs. When silent synapses are gradually replaced by AMPAR-containing synapses, the dominant form of cortical network activity is driven by depolarizing GABA (Ben-Ari et al., 1997; Ben-Ari, 2002; Allène et al., 2008). This suggests that depolarizing GABA may provide the neural activity for the unsilencing of silent synapses (Ben-Ari et al., 1997; Ben-Ari, 2002). Indeed GABA receptor-mediated depolarization is required for the unsilencing of silent synapses in the developing neocortex (Wang and Kriegstein, 2008) and during the development of adult-generated dentate granule cells (Chancey et al., 2013), but silent synapses can also be unsilenced by seizure activity (Zhou et al., 2011; Sun et al., 2018).

Besides the depolarizing action of GABA, neuromodulators which depolarize SPNs could also play a role in unsilencing silent connections. SPNs have both ACh (Hanganu et al., 2002; Hanganu and Luhmann, 2004) as well as serotonin receptors (Kanold and Luhmann, 2010; Liao and Lee, 2011, 2014) which can act depolarizing. Thus, the actions of these neuromodulators could contribute to the functional unsilencing of silent connections to SPNs.

Thus, in summary, there are multiple non-exclusive sources of depolarizing inputs that might individually or jointly act in unsilencing silent connections to SPNs.

## What Are Silent Synapses Good for? What do They Tell us?

NMDAR-mediated connections are not only present in the developing mammalian cerebral cortex but also in other structures. Thus, the computational roles of such connections might be similar. First, it is well-established that NMDAR-only mediated synaptic connections can function as a coincidence detector. The activation of NMDAR not only requires glutamate binding but also membrane depolarization to remove the Mg^2+^ from the channel pore. One role of a coincidence detector is to enable the refinement of topographic connections as illustrated in the retinogeniculate system. During early development, structured spontaneous activity is present in the retina (Galli and Maffei, 1988; Meister et al., 1991; Feller et al., 1996; Firth et al., 2005) and competition between the two eyes drives retino-geniculate (Penn et al., 1998) and retino-collicular (Shah and Crair, 2008) segregation. Blockade of NMDA signaling alters the segregation of retinal ganglion afferents in both the lateral geniculate nucleus as well as the optic tectum (Hahm et al., 1991; Ruthazer et al., 2003; Ben Fredj et al., 2010).

However, the functional contribution of silent synapses to these processes remains unclear possibly because few retino-geniculate connections are truly “silent” (Hohnke et al., 2000). Moreover, it seems that at least in the retino-geniculate system, silent synapses peak after the increase in sensory-driven activity, thus might play a role at slightly later stages in development (Lu and Constantine-Paton, 2004).

A second potential role of NMDAR-only mediated synaptic connections is to integrate multiple subthreshold EPSCs over time and improve the synchrony between different cells due to a longer activation time scale. This is consistent with the role of NMDARs in supporting synchronized oscillations in the rodent somatosensory cortex (Dupont et al., 2006; Yang et al., 2009).

A third role of silent synapses has been shown recently in the mammalian retina to enhance motion processing by enabling differential processing of a single source of glutamate by two cells (Sethuramanujam et al., 2017).

Given the fact that activity in developing networks is slow and adapting, it seems likely that silent synapses on SPNs are involved in associating activity over longer time scales but do not enhance processing of stimuli, activity, or coincidence on fast time scales.

Moreover, since silent synapses can be unsilenced by correlated activity thus, by extension, the pattern of unsilenced, AMPAR- and NMDAR-containing, connections are in effect a readout of the past amount of correlated activity of inputs to a particular neuron. However, since unsilencing requires coincidence, the pattern of unsilenced neurons might not necessarily reflect the amount of activity *per se*. Thus, the presence of AMPAR-containing synapses on connections from deep layers indicates that those layers were likely correlated with SPNs. The emergence of AMPAR-containing synapses from L4 to SPNs after ear opening in the auditory cortex (Meng et al., 2014) indicates that L4 activity likely was correlated with SPN activity just before ear opening (Figure 1B). Such an interpretation would be consistent with the increased evoked correlated activity between SPNs and L4 at similar stages of development in ferret (Wess et al., 2017). Interestingly, just before ear opening, correlated activity is largest in the beta band, possibly indicating that such activity might be effective in unsilencing of synapses. Moreover, the spatial pattern of unsilenced synapses from the cortical plate shows a columnar pattern indicating synchronous activity across a cortical column consistent with microelectrode studies (Yang et al., 2009, 2013; Colonnese and Khazipov, 2010; Minlebaev et al., 2011).

## SPN Might Enable the Generation of Normal Activity Patterns in the Developing Cortex

The existence of abundant NMDAR-only silent synapses in SPNs and other cortical neurons suggests that most excitatory activity during early development is mediated by NMDARs. Accordingly, cortical network activity *in vitro* during the first postnatal week can be almost completely blocked by application of the NMDAR antagonist AP5 (Allène et al., 2008). These results suggest that NMDAR-only “silent” synapses are in fact not “silent” but conduct current and regulate cortical network activity. Thus, NMDAR-only synapses are not truly “silent” but represent a unique early developmental mode of activity generation and propagation.

The electrical activity in the developing brain and especially the cortex is dominated by oscillations. These oscillations can be present spontaneously, that is without any overt external trigger, or they can be evoked by sensory stimuli such as light flashes, motor twitches, or sounds (Khazipov et al., 2004; Hanganu et al., 2006; Marcano-Reik and Blumberg, 2008; Yang et al., 2009; Colonnese and Khazipov, 2010; Chipaux et al., 2013; Blumberg et al., 2015). Moreover, spontaneous activity can be generated endogenously in the cortex (Garaschuk et al., 2000; Adelsberger et al., 2005; Siegel et al., 2012) or even in cortical slice culture (Stewart and Plenz, 2008) as well as be reflective of spontaneously generated activity in the sensory periphery e.g., the retina or cochlea (Galli and Maffei, 1988; Meister et al., 1991; Feller et al., 1996; Firth et al., 2005; Tritsch et al., 2007; Siegel et al., 2012; Wang and Bergles, 2015).

These activity patterns are complex and are comprised of fast nested oscillatory patterns, the so-called “spindles,” and slower transients, “SATs” (Vanhatalo et al., 2002; Tolonen et al., 2007; Benders et al., 2015). Centrally and peripherally generated spontaneous activity show different amounts of synchronizing, wave progression, and developmental profile (Siegel et al., 2012). These early activity patterns are thought to be crucially required for the functional maturation of cortical circuits as they enable high local synchrony of activity (Vanhatalo and Kaila, 2006; Tolonen et al., 2007; Kilb et al., 2011; Siegel et al., 2012; Khazipov et al., 2013; Winnubst et al., 2015; Yang et al., 2016; Luhmann and Khazipov, 2018). Many activity patterns can be replicated using *in vitro* preparations indicating that at minimum cortical circuits and thalamocortical inputs are required (Sun and Luhmann, 2007; Sun et al., 2010). The origin of early cortical oscillations is not known but likely involve the complex interplay of ascending thalamocortical and descending corticothalamic circuits (Yang et al., 2013, 2016; Murata and Colonnese, 2016). Due to their location at the center of thalamocortical and corticothalamic circuits, SPNs can influence cortical activity patterns. Indeed, electrical stimulation of SPNs in an *in vitro* preparation could cause oscillations (Sun and Luhmann, 2007) and removal of SPNs *in vitro* abolishes oscillations (Dupont et al., 2006). Further evidence for the crucial role of SPNs in the development of cortical oscillations came from selective ablation studies (Tolner et al., 2012). These *in vivo* ablation studies showed that SPNs are required for both spontaneous and sensory-evoked oscillations (Tolner et al., 2012). Together, these observations point to a key role of SPNs in the generation or transmission of cortical oscillations. Since, during the time when spontaneous activity is present, many connections to SPNs are NMDAR-only, this suggests that NMDAR-only synapses on SPNs might play a key role in controlling cortical oscillations. Indeed, the synchronized oscillations in developing somatosensory cortex are mediated through NMDARs (Dupont et al., 2006; Minlebaev et al., 2009; Yang et al., 2009).

## SPNs Might Conditionally Integrate Ascending and Intracortical Activity *via* Silent Synapses

The above reflections and the emerging connectivity diagram suggest that the presence of abundant silent and non-silent synapses on SPNs enables SPNs to play important associative roles during development. As mentioned above, SPNs are heterogenous and can functionally be classified into two main groups based on their amount of L4 input but which can further be subdivided based on the amount of intralaminar connectivity which in auditory cortex likely reflects integration across the frequency axis (Meng et al., 2014). One group of SPNs (Figure 2A groups 1 and 3) does not receive L4 inputs and thus primarily depends on feed-forward inputs. The second group (Figure 2A groups 2 and 4) does not only integrate feed-forward thalamocortical inputs from MGB but also receives significant feedback inputs from L4. Thus, both feed-forward and feed-back inputs are affected by the resting potential. Both groups of SPNs can be further subdivided based on the amount of lateral intralaminar integration. Given the heterogeneity, the role of silent connections likely differs between these subgroups of SPNs.

On one hand, SPNs receive early spontaneous and sensory-evoked inputs *via* the thalamus and on the other hand, they receive early cortical inputs. While sensory stimulation can lead to activity in the matching cortical area (e.g., sounds in auditory cortex or touch in somatosensory cortex), other sources of intracortical activity can also be present such as activity, e.g., multisensory, originating in other cortical areas or intrinsically generated non-sensory evoked activity (Garaschuk et al., 2000; Adelsberger et al., 2005; Khazipov and Luhmann, 2006; Siegel et al., 2012). Thus, by virtue of their position between ascending thalamic circuits and cortical circuits, SPNs can potentially associate ascending and intracortical activity. Moreover, the presence of NMDARs, and not AMPARs, allows SPNs to directly gate and associate cortical activity with ascending thalamic information (Figure 2B). This is due to the fact that NMDARs will not signal unless SPNs are depolarized. Ascending thalamic input can depolarize SPNs and thus remove the Mg^2+^ block from NMDARs. If sufficient cortical activity is present at the same time then cortico-SP synapses will signal, enhancing the SPN response. Since SPNs innervate L4 and beyond, a temporal coincidence of cortical activity with the ascending activity will boost the ascending thalamic signal. Moreover, because SPNs also project to the thalamus (Viswanathan et al., 2017; Hoerder-Suabedissen et al., 2018), which is involved in generating oscillatory activity, boosted SPN activity has the potential to influence the generation of thalamocortical oscillations. Thus, over time this scenario likely leads to an association or synchronization of both sources of activity.

The changing presence of silent synapses changes the association between thalamic and cortical inputs. The addition of AMPARs at intracortical inputs at later ages transforms the initial conditional or gating association of thalamic and cortical inputs into an additive association (Figure 2B).

Besides ascending thalamic inputs other sources of activity can activate silent synapses and change the associative properties of SPNs. Because developing SPNs might have a slightly depolarized resting potential (Zhao et al., 2009), NMDAR-mediated synapses can potentially be activated by slight depolarizations from the resting potentials. In particular, the GABAR-mediated inputs to SPNs can be depolarizing due to high intracellular Cl^−^ level at early ages (Luhmann et al., 2016). Thus, depolarizing GABA might be able to activate the silent synapses on SPNs. Besides, since developing SPNs tend to have higher membrane resistances (Zhao et al., 2009), which leads to longer time constants, weak depolarizing inputs could be integrated in SPNs and potentially activate the NMDAR-only mediated inputs.

Thus, the existence of silent synapses from spatially specific presynaptic cells suggests a very interesting hypothesis on the functional topology of the developing cortical circuit: the inter- and intra-columnar connections involving SPNs could be dynamic (Figure 2C). In other words, silent synapses can enable SPNs to integrate inputs from either close by or farther away in a state-dependent manner.

Since a crucial variable in acutely unsilencing silent synapses is the resting membrane potential, relatively slow acting neuromodulatory inputs could play a major role in determining the amount of integration taking place in SPNs. As mentioned above, SPNs have ACh as well as serotonin receptors (Hanganu et al., 2002, 2009; Hanganu and Luhmann, 2004; Kanold and Luhmann, 2010; Liao and Lee, 2011, 2014). Moreover, layer 6b neurons in adult, a subset of which represents surviving SPNs (Marx et al., 2017), are modulated by neurotensin controlling wakefulness (Case and Broberger, 2018). It is possible that SPNs are modulated by neuropeptides even at early ages and that their function varies thru the sleep-wake cycle due to differential recruitment of local AMPAR-mediated vs. distal NMDAR-only mediated inputs.

## Disruption of SPN in Clinical Conditions and Potential Role of Silent Synapses

**Figure 3:**
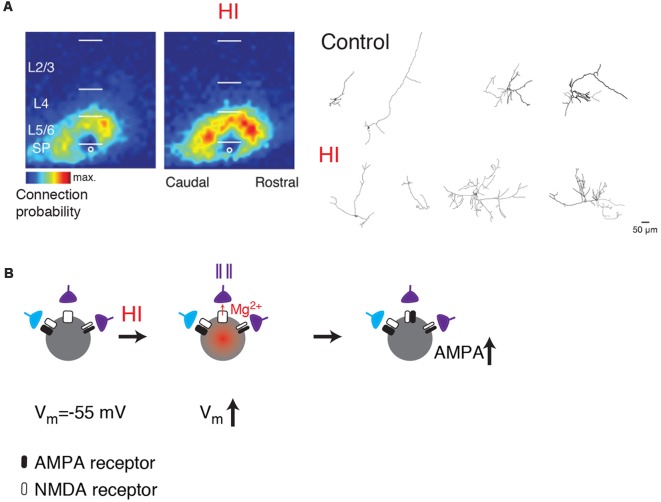
**(A)** SPNs show functional and morphological hyperconnectivity after hypoxic-ischemic (HI; from Sheikh et al., 2019; no copyright permission as required for use of this image). Shown are LSPS maps of connection probability and neurolucida reconstructions.** (B)** Cartoon illustrating how depolarization caused by HI can lead to unsilencing of synapses on SPNs (middle). If presynaptic cells are active (indicated by spike train), the coincidence between presynaptic activity and postsynaptic depolarization might result in a subsequent increase in AMPARs (right, black).

Given the central position of SPNs, disruption of SPNs can alter many aspects of cortical development (Ghosh et al., 1990; Ghosh and Shatz, 1992; McConnell et al., 1994; Kanold et al., 2003; Kanold and Shatz, 2006; Tolner et al., 2012). Because of their early maturation and connectivity, SPNs are also susceptible to injury and resulting malfunction. For example, neonatal hypoxic-ischemic (HI) injuries in animals can lesion SPNs or in milder forms cause hyperconnectivity of SPNs (McQuillen et al., 2003; Mikhailova et al., 2017; Sheikh et al., 2019; Figure 3A). Such injuries are associated with altered cortical function and plasticity (Failor et al., 2010; Ranasinghe et al., 2015) similar to those observed after SPN lesion (Kanold et al., 2003; Kanold and Shatz, 2006; Tolner et al., 2012) suggesting that SPN damage after the HI injury might be leading to the downstream effects in the cortical plate.

In humans, SPN generation begins around the fifth postconceptional week and fully formed between the 20–26th week (Kanold and Luhmann, 2010). Environmental insults that occur during the first trimester can increase the risk of autism. For instance, valproate (VPA) exposure during the first trimester correlates negatively with language outcome in children (Nadebaum et al., 2011). In mice, subplate birth occurs between E11–13, and full formation of subplate by E14 (Kanold and Luhmann, 2010). Although this is outside of the first “trimester,” maternal VPA exposure during this narrow time window results in autistic phenotypes in the offspring (Mabunga et al., 2015; Nicolini and Fahnestock, 2018). Recent *in vitro* studies in rodents have shown that such prenatal VPA exposure causes miswiring of circuits to SPNs (Nagode et al., 2017). While these circuit changes were detected shortly after birth (Nagode et al., 2017), earlier ages were not studied, thus it is possible that even earlier effects of VPA on SPNs are present. Together, these studies in animal models suggest that SPNs might form a key element in the progression from prenatal insult to disease phenotype in multiple neurodevelopmental disorders.

Because subplate disappears before the onset of ASD symptoms, no direct link has been made between subplate and ASD in humans. However, one of the more intriguing indirect links is the relatively consistent finding of a blurred gray-white matter boundary in the brains of ASD individuals (Avino and Hutsler, 2010; Hutsler and Casanova, 2016), possibly due to an increase in the number of interstitial neurons in the superficial white matter. Interstitial neurons are believed to be remnants of the fetal subplate (Chun and Shatz, 1989; Reep, 2000; Torres-Reveron and Friedlander, 2007; Suárez-Solá et al., 2009; Judaš et al., 2010), and their overabundance could reflect dysfunctional apoptotic mechanisms in ASD (Courchesne and Pierce, 2005; Avino and Hutsler, 2010; Courchesne et al., 2011; McFadden and Minshew, 2013). Autistic patients also show an altered columnar structure in frontal cortex (Buxhoeveden et al., 2006) and patches of disorganization are seen in L4 and L5 of the prefrontal cortex in children with autism (Stoner et al., 2014). Given the putative role of subplate in setting up protomaps of cortical organization (O’Leary and Borngasser, 2006; O’Leary et al., 2007; Wess et al., 2017; Kanold, 2019) and given that alterations are present in the target layers of SPNs, these observations are consistent with a potential role of early SPN dysfunction in the emergence of ASDs. However, the ultimate test of this possibility would require to reverse or to prevent the changes in the SPNs, which in turn could lead to treatment and prevention approaches in humans. The consequences of increased “adult subplate” or interstitial cells on circuit function are not known.

Additional indirect evidence points to a role of SPNs in ASDs. Expression profiling has identified an enrichment in subplate genes for association with ASDs (Hoerder-Suabedissen et al., 2013) but there are additional potential links between subplate damage and ASDs. Hypoxia/ischemia preferentially damages SPNs in rodents (McQuillen et al., 2003; Mikhailova et al., 2017; Sheikh et al., 2019). Birth injuries or preterm labor can lead to hypoxic situations, and both of these have been shown to increase the risk of ASD (reviewed in Rennie et al., 2007; Agrawal et al., 2018; Rogers et al., 2018). Perinatal anoxia in rats also causes ASD phenotypes (Laviola et al., 2004). Importantly, preterm birth is also associated with an increased risk of ASD, and this might be due to an increase in the incidence of pre-eclampsia in mother of preterm infants, among other things (Buchmayer et al., 2009).

Thus, while direct evidence of SPN disruptions in ASDs is currently lacking, multiple lines of evidence suggest that understanding SPN function and susceptibility will be important in understanding the etiology of many neurodevelopmental disorders. One common thread is the vulnerability of SPNs to hypoxia/ischemia. While the acute effects of hypoxic/ischemic injuries on SPNs are unknown, anoxia can induce depolarization in cortical pyramidal cells (Luhmann et al., 1993; Rosen and Morris, 1994; O’Reilly and Haddad, 1996; Pisani et al., 1997). Thus, hypoxia might be able to “unsilence” silent synapses onto SPNs leading to an increased co-activation of presynaptic neurons and postsynaptic SPNs (Figure 3B). Such co-activation would be expected to result in increased connectivity, which is experimentally seen after HI (Sheikh et al., 2019). Indeed, *in vitro* studies showed that combined oxygen and glucose deprivation depolarizes SPNs (Albrecht et al., 2005). Moreover, hypoxia-induced seizures can diminish silent synapses in hippocampus consistent with such a scenario (Zhou et al., 2011). Thus, activation of silent synapses on SPNs by hypoxia or other insults might be a key step in linking early insults to later circuit dysfunctions.

## Conclusion and Open Questions

While SPNs as a population of neurons are coming into clearer view, many unknowns remain. SPNs as a group are both the target of initial thalamic inputs but also receive extensive cortical input. In particular, there is an extensive connectivity of SPNs with the cortical plate mediated by NMDAR-only containing “silent” synapses at the earliest ages. These synapses are very sensitive to the resting potential of the cell, thus subtle modulation of the resting potential can modulate their activity and thus their ability to integrate inputs. Activation of silent synapses on SPNs by hypoxia or other insults might be the initial key step in a cascade of events leading to the manifestation of neurodevelopmental disorders. Therefore, interventions that limit the amount of depolarization or NMDAR signaling might be effective in preventing this chain of events. Indeed, hypothermia has been shown to be effective in reducing the effects of hypoxia on cortical neurons (Hiramatsu et al., 1993; Rosen and Morris, 1994) and improves clinical outcomes (Gunn and Bennet, 2008; Jacobs et al., 2013; Papile et al., 2014).

It is becoming clear that multiple classes of SPNs exist, but the functional roles of the subclasses are unknown. Moreover, given that SPNs are present in all cortical areas, it is likely that there are functional differences between SPNs in different cortical areas. This has not been explored at all. Most importantly, the role of SPNs in development has only been assessed in primary sensory areas, thus important roles of SPNs in other areas, e.g., frontal areas, that might relate to a potential role of SPNs in neurodevelopmental disorders is lacking. SPNs are present in all mammalian species but given that the size of the subplate zone varies between species, it is likely that there are species-dependent differences in SPN organization and possibly function. Such differences have not been elucidated. In particular, the function of SPNs in primates has not been studied. Thus, future studies need to address the functional diversity of SPN within but also across species. Moreover, there is growing evidence of SPN dysfunction in multiple neurodevelopmental disorders. What is lacking is direct functional evidence of SPN disruption in human patients at the relevant early developmental ages. Moreover, while SPNs have been studied in primary sensory areas the phenotypes of many neurodevelopmental disorders have been most prevalent in frontal areas leaving open the possibility that dysfunctions might be more widespread or that a selective vulnerability exists for circuits in frontal areas. Functional imaging or EEG methods might be able to provide such evidence.

## Author Contributions

All authors made direct and intellectual contribution to the work and approved it for publication. PK wrote the manuscript with contributions from XM and RD.

## Conflict of Interest Statement

The authors declare that the research was conducted in the absence of any commercial or financial relationships that could be construed as a potential conflict of interest.

## References

[B1] Adelsberger H. Garaschuk O. Konnerth A. (2005). Cortical calcium waves in resting newborn mice. Nat. Neurosci. 8, 988–990. 10.1038/nn1502 16007081

[B2] Agrawal S. Rao S. C. Bulsara M. K. Patole S. K. (2018). Prevalence of autism spectrum disorder in preterm infants: a meta-analysis. Pediatrics 142:e20180134. 10.1542/peds.2018-0134 30076190

[B3] Albrecht J. Hanganu I. L. Heck N. Luhmann H. J. (2005). Oxygen and glucose deprivation induces major dysfunction in the somatosensory cortex of the newborn rat. Eur. J. Neurosci. 22, 2295–2305. 10.1111/j.1460-9568.2005.04398.x 16262667

[B4] Allène C. Cattani A. Ackman J. B. Bonifazi P. Aniksztejn L. Ben-Ari Y.. (2008). Sequential generation of two distinct synapse-driven network patterns in developing neocortex. J. Neurosci. 28, 12851–12863. 10.1523/JNEUROSCI.3733-08.2008 19036979 PMC6671804

[B5] Anastasiades P. G. Butt S. J. (2012). A role for silent synapses in the development of the pathway from layer 2/3 to 5 pyramidal cells in the neocortex. J. Neurosci. 32, 13085–13099. 10.1523/JNEUROSCI.1262-12.2012 22993426 PMC6621489

[B6] Antonini A. Shatz C. J. (1990). Relation between putative transmitter phenotypes and connectivity of subplate neurons during cerebral cortical development. Eur. J. Neurosci. 2, 744–761. 10.1111/j.1460-9568.1990.tb00465.x 12106275

[B7] Ashby M. C. Isaac J. T. (2011). Maturation of a recurrent excitatory neocortical circuit by experience-dependent unsilencing of newly formed dendritic spines. Neuron 70, 510–521. 10.1016/j.neuron.2011.02.057 21555076 PMC3092126

[B8] Avino T. A. Hutsler J. J. (2010). Abnormal cell patterning at the cortical gray-white matter boundary in autism spectrum disorders. Brain Res. 1360, 138–146. 10.1016/j.brainres.2010.08.091 20816758

[B10] Ben-Ari Y. (2002). Excitatory actions of gaba during development: the nature of the nurture. Nat. Rev. Neurosci. 3, 728–739. 10.1038/nrn920 12209121

[B11] Ben-Ari Y. Khazipov R. Leinekugel X. Caillard O. Gaiarsa J. L. (1997). GABA_A_, NMDA and AMPA receptors: a developmentally regulated ‘ménage à trois’. Trends Neurosci. 20, 523–529. 10.1016/s0166-2236(97)01147-8 9364667

[B9] Ben Fredj N. Hammond S. Otsuna H. Chien C. B. Burrone J. Meyer M. P. (2010). Synaptic activity and activity-dependent competition regulates axon arbor maturation, growth arrest, and territory in the retinotectal projection. J. Neurosci. 30, 10939–10951. 10.1523/JNEUROSCI.1556-10.2010 20702722 PMC6634700

[B12] Benders M. J. Palmu K. Menache C. Borradori-Tolsa C. Lazeyras F. Sizonenko S.. (2015). Early brain activity relates to subsequent brain growth in premature infants. Cereb. Cortex 25, 3014–3024. 10.1093/cercor/bhu097 24867393

[B13] Blumberg M. S. Coleman C. M. Sokoloff G. Weiner J. A. Fritzsch B. McMurray B. (2015). Development of twitching in sleeping infant mice depends on sensory experience. Curr. Biol. 25, 656–662. 10.1016/j.cub.2015.01.022 25702578 PMC4348337

[B14] Bruno R. M. (2011). Synchrony in sensation. Curr. Opin. Neurobiol. 21, 701–708. 10.1016/j.conb.2011.06.003 21723114 PMC3191231

[B15] Buchmayer S. Johansson S. Johansson A. Hultman C. M. Sparén P. Cnattingius S. (2009). Can association between preterm birth and autism be explained by maternal or neonatal morbidity? JAMA 124, e817–e825. 10.1542/peds.2008-3582 19841112

[B16] Butovas S. Hormuzdi S. G. Monyer H. Schwarz C. (2006). Effects of electrically coupled inhibitory networks on local neuronal responses to intracortical microstimulation. J. Neurophysiol. 96, 1227–1236. 10.1152/jn.01170.2005 16837655

[B17] Butts D. A. Kanold P. O. (2010). The applicability of spike time dependent plasticity to development. Front. Synaptic Neurosci. 2:30. 10.3389/fnsyn.2010.00030 21423516 PMC3059702

[B18] Buxhoeveden D. P. Semendeferi K. Buckwalter J. Schenker N. Switzer R. Courchesne E. (2006). Reduced minicolumns in the frontal cortex of patients with autism. Neuropathol. Appl. Neurobiol. 32, 483–491. 10.1111/j.1365-2990.2006.00745.x 16972882

[B19] Case L. Broberger C. (2018). Neurotensin broadly recruits inhibition via white matter neurons in the mouse cerebral cortex: synaptic mechanisms for decorrelation. Cereb. Cortex 28, 2711–2724. 10.1093/cercor/bhx149 28981614

[B20] Chancey J. H. Adlaf E. W. Sapp M. C. Pugh P. C. Wadiche J. I. Overstreet-Wadiche L. S. (2013). GABA depolarization is required for experience-dependent synapse unsilencing in adult-born neurons. J. Neurosci. 33, 6614–6622. 10.1523/JNEUROSCI.0781-13.2013 23575858 PMC3657840

[B21] Chipaux M. Colonnese M. T. Mauguen A. Fellous L. Mokhtari M. Lezcano O.. (2013). Auditory stimuli mimicking ambient sounds drive temporal “delta-brushes” in premature infants. PLoS One 8:e79028. 10.1371/journal.pone.0079028 24244408 PMC3823968

[B22] Chun J. J. Shatz C. J. (1989). Interstitial cells of the adult neocortical white matter are the remnant of the early generated subplate neuron population. J. Comp. Neurol. 282, 555–569. 10.1002/cne.902820407 2566630

[B23] Clancy B. Cauller L. J. (1999). Widespread projections from subgriseal neurons (layer VII) to layer I in adult rat cortex. J. Comp. Neurol. 407, 275–286. 10.1002/(sici)1096-9861(19990503)407:2<275::aid-cne8>3.0.co;2-0 10213095

[B24] Colonnese M. T. Khazipov R. (2010). “Slow activity transients” in infant rat visual cortex: a spreading synchronous oscillation patterned by retinal waves. J. Neurosci. 30, 4325–4337. 10.1523/JNEUROSCI.4995-09.2010 20335468 PMC3467103

[B25] Committee on Fetus and Newborn Papile L. A. Baley J. E. Benitz W. Cummings J. Carlo W. A.. (2014). Hypothermia and neonatal encephalopathy. Pediatrics 133, 1146–1150. 10.1542/peds.2014-0899 24864176

[B26] Connors B. W. Long M. A. (2004). Electrical synapses in the mammalian brain. Annu. Rev. Neurosci. 27, 393–418. 10.1146/annurev.neuro.26.041002.131128 15217338

[B27] Constantinople C. M. Bruno R. M. (2013). Deep cortical layers are activated directly by thalamus. Science 340, 1591–1594. 10.1126/science.1236425 23812718 PMC4203320

[B28] Courchesne E. Mouton P. R. Calhoun M. E. Semendeferi K. Ahrens-Barbeau C. Hallet M. J.. (2011). Neuron number and size in prefrontal cortex of children with autism. JAMA 306, 2001–2010. 10.1001/jama.2011.1638 22068992

[B29] Courchesne E. Pierce K. (2005). Brain overgrowth in autism during a critical time in development: implications for frontal pyramidal neuron and interneuron development and connectivity. Int. J. Dev. Neurosci. 23, 153–170. 10.1016/j.ijdevneu.2005.01.003 15749242

[B30] Cruikshank S. J. Landisman C. E. Mancilla J. G. Connors B. W. (2005). Connexon connexions in the thalamocortical system. Prog. Brain Res. 149, 41–57. 10.1016/s0079-6123(05)49004-4 16226575

[B31] Deans M. R. Gibson J. R. Sellitto C. Connors B. W. Paul D. L. (2001). Synchronous activity of inhibitory networks in neocortex requires electrical synapses containing connexin36. Neuron 31, 477–485. 10.1016/s0896-6273(01)00373-7 11516403

[B32] Deng R. Kao J. P. Y. Kanold P. O. (2017). Distinct translaminar glutamatergic circuits to GABAergic interneurons in the neonatal auditory cortex. Cell Rep. 19, 1141–1150. 10.1016/j.celrep.2017.04.044 28494864 PMC5652063

[B33] Dupont E. Hanganu I. L. Kilb W. Hirsch S. Luhmann H. J. (2006). Rapid developmental switch in the mechanisms driving early cortical columnar networks. Nature 439, 79–83. 10.1038/nature04264 16327778

[B34] Durand G. M. Kovalchuk Y. Konnerth A. (1996). Long-term potentiation and functional synapse induction in developing hippocampus. Nature 381, 71–75. 10.1038/381071a0 8609991

[B35] Failor S. Nguyen V. Darcy D. P. Cang J. Wendland M. F. Stryker M. P.. (2010). Neonatal cerebral hypoxia-ischemia impairs plasticity in rat visual cortex. J. Neurosci. 30, 81–92. 10.1523/JNEUROSCI.5656-08.2010 20053890 PMC2822440

[B36] Feller M. B. Wellis D. P. Stellwagen D. Werblin F. S. Shatz C. J. (1996). Requirement for cholinergic synaptic transmission in the propagation of spontaneous retinal waves. Science 272, 1182–1187. 10.1126/science.272.5265.1182 8638165

[B37] Finney E. M. Stone J. R. Shatz C. J. (1998). Major glutamatergic projection from subplate into visual cortex during development. J. Comp. Neurol. 398, 105–118. 10.1002/(SICI)1096-9861(19980817)398:1<105::AID-CNE7>3.0.CO;2-5 9703030

[B38] Firth S. I. Wang C. T. Feller M. B. (2005). Retinal waves: mechanisms and function in visual system development. Cell Calcium 37, 425–432. 10.1016/j.ceca.2005.01.010 15820390

[B39] Friauf E. McConnell S. K. Shatz C. J. (1990). Functional synaptic circuits in the subplate during fetal and early postnatal development of cat visual cortex. J. Neurosci. 10, 2601–2613. 10.1523/JNEUROSCI.10-08-02601.1990 2388080 PMC6570284

[B40] Friauf E. Shatz C. J. (1991). Changing patterns of synaptic input to subplate and cortical plate during development of visual cortex. J. Neurophysiol. 66, 2059–2071. 10.1152/jn.1991.66.6.2059 1812236

[B41] Fukuda T. Kosaka T. Singer W. Galuske R. A. (2006). Gap junctions among dendrites of cortical GABAergic neurons establish a dense and widespread intercolumnar network. J. Neurosci. 26, 3434–3443. 10.1523/JNEUROSCI.4076-05.2006 16571750 PMC6673861

[B42] Funahashi R. Maruyama T. Yoshimura Y. Komatsu Y. (2013). Silent synapses persist into adulthood in layer 2/3 pyramidal neurons of visual cortex in dark-reared mice. J. Neurophysiol. 109, 2064–2076. 10.1152/jn.00912.2012 23343903

[B43] Galarreta M. Hestrin S. (1999). A network of fast-spiking cells in the neocortex connected by electrical synapses. Nature 402, 72–75. 10.1038/47029 10573418

[B44] Galarreta M. Hestrin S. (2001). Electrical synapses between GABA-releasing interneurons. Nat. Rev. Neurosci. 2, 425–433. 10.1038/35077566 11389476

[B45] Galarreta M. Hestrin S. (2002). Electrical and chemical synapses among parvalbumin fast-spiking GABAergic interneurons in adult mouse neocortex. Proc. Natl. Acad. Sci. U S A 99, 12438–12443. 10.1073/pnas.192159599 12213962 PMC129463

[B46] Galli L. Maffei L. (1988). Spontaneous impulse activity of rat retinal ganglion cells in prenatal life. Science 242, 90–91. 10.1126/science.3175637 3175637

[B47] Garaschuk O. Linn J. Eilers J. Konnerth A. (2000). Large-scale oscillatory calcium waves in the immature cortex. Nat. Neurosci. 3, 452–459. 10.1038/74823 10769384

[B48] Ghosh A. Antonini A. McConnell S. K. Shatz C. J. (1990). Requirement for subplate neurons in the formation of thalamocortical connections. Nature 347, 179–181. 10.1038/347179a0 2395469

[B49] Ghosh A. Shatz C. J. (1992). Involvement of subplate neurons in the formation of ocular dominance columns. Science 255, 1441–1443. 10.1126/science.1542795 1542795

[B50] Gibson J. R. Beierlein M. Connors B. W. (1999). Two networks of electrically coupled inhibitory neurons in neocortex. Nature 402, 75–79. 10.1038/47035 10573419

[B51] Gunn A. J. Bennet L. (2008). Brain cooling for preterm infants. Clin. Perinatol. 35, 735–748, vi–vii. 10.1016/j.clp.2008.07.012 19026337

[B52] Hahm J. O. Langdon R. B. Sur M. (1991). Disruption of retinogeniculate afferent segregation by antagonists to NMDA receptors. Nature 351, 568–570. 10.1038/351568a0 1675433

[B53] Han K. S. Cooke S. F. Xu W. (2017). Experience-dependent equilibration of AMPAR-mediated synaptic transmission during the critical period. Cell Rep. 18, 892–904. 10.1016/j.celrep.2016.12.084 28122240

[B54] Hanganu I. L. Ben-Ari Y. Khazipov R. (2006). Retinal waves trigger spindle bursts in the neonatal rat visual cortex. J. Neurosci. 26, 6728–6736. 10.1523/JNEUROSCI.0752-06.2006 16793880 PMC6673818

[B55] Hanganu I. L. Kilb W. Luhmann H. J. (2002). Functional synaptic projections onto subplate neurons in neonatal rat somatosensory cortex. J. Neurosci. 22, 7165–7176. 10.1523/JNEUROSCI.22-16-07165.2002 12177212 PMC6757868

[B56] Hanganu I. L. Luhmann H. J. (2004). Functional nicotinic acetylcholine receptors on subplate neurons in neonatal rat somatosensory cortex. J. Neurophysiol. 92, 189–198. 10.1152/jn.00010.2004 14999055

[B57] Hanganu I. L. Okabe A. Lessmann V. Luhmann H. J. (2009). Cellular mechanisms of subplate-driven and cholinergic input-dependent network activity in the neonatal rat somatosensory cortex. Cereb. Cortex 19, 89–105. 10.1093/cercor/bhn061 18440948

[B58] Hanse E. Seth H. Riebe I. (2013). AMPA-silent synapses in brain development and pathology. Nat. Rev. Neurosci. 14, 839–850. 10.1038/nrn3642 24201185

[B59] Henschke J. U. Oelschlegel A. M. Angenstein F. Ohl F. W. Goldschmidt J. Kanold P. O.. (2018). Early sensory experience influences the development of multisensory thalamocortical and intracortical connections of primary sensory cortices. Brain Struct. Funct. 223, 1165–1190. 10.1007/s00429-017-1549-1 29094306 PMC5871574

[B60] Higashi S. Hioki K. Kurotani T. Kasim N. Molnár Z. (2005). Functional thalamocortical synapse reorganization from subplate to layer IV during postnatal development in the reeler-like mutant rat (*shaking rat Kawasaki*). J. Neurosci. 25, 1395–1406. 10.1523/JNEUROSCI.4023-04.2005 15703393 PMC6725983

[B61] Higashi S. Molnár Z. Kurotani T. Toyama K. (2002). Prenatal development of neural excitation in rat thalamocortical projections studied by optical recording. Neuroscience 115, 1231–1246. 10.1016/s0306-4522(02)00418-9 12453494

[B62] Higo S. Udaka N. Tamamaki N. (2007). Long-range GABAergic projection neurons in the cat neocortex. J. Comp. Neurol. 503, 421–431. 10.1002/cne.21395 17503478

[B63] Hiramatsu K. Kassell N. F. Lee K. S. (1993). Thermal sensitivity of hypoxic responses in neocortical brain slices. J. Cereb. Blood Flow Metab. 13, 395–401. 10.1038/jcbfm.1993.53 8478398

[B64] Hoerder-Suabedissen A. Hayashi S. Upton L. Nolan Z. Casas-Torremocha D. Grant E.. (2018). Subset of cortical layer 6b neurons selectively innervates higher order thalamic nuclei in mice. Cereb. Cortex 28, 1882–1897. 10.1093/cercor/bhy036 29481606 PMC6018949

[B65] Hoerder-Suabedissen A. Oeschger F. M. Krishnan M. L. Belgard T. G. Wang W. Z. Lee S.. (2013). Expression profiling of mouse subplate reveals a dynamic gene network and disease association with autism and schizophrenia. Proc. Natl. Acad. Sci. U S A 110, 3555–3560. 10.1073/pnas.1218510110 23401504 PMC3587197

[B66] Hoerder-Suabedissen A. Wang W. Z. Lee S. Davies K. E. Goffinet A. M. Rakić S.. (2009). Novel markers reveal subpopulations of subplate neurons in the murine cerebral cortex. Cereb. Cortex 19, 1738–1750. 10.1093/cercor/bhn195 19008461

[B67] Hohnke C. D. Oray S. Sur M. (2000). Activity-dependent patterning of retinogeniculate axons proceeds with a constant contribution from AMPA and NMDA receptors. J. Neurosci. 20, 8051–8060. 10.1523/JNEUROSCI.20-21-08051.2000 11050126 PMC6772753

[B68] Hutsler J. J. Casanova M. F. (2016). Review: cortical construction in autism spectrum disorder: columns, connectivity and the subplate. Neuropathol. Appl. Neurobiol. 42, 115–134. 10.1111/nan.12227 25630827

[B69] Isaac J. T. (2003). Postsynaptic silent synapses: evidence and mechanisms. Neuropharmacology 45, 450–460. 10.1016/s0028-3908(03)00229-6 12907306

[B70] Isaac J. T. Crair M. C. Nicoll R. A. Malenka R. C. (1997). Silent synapses during development of thalamocortical inputs. Neuron 18, 269–280. 10.1016/s0896-6273(00)80267-6 9052797

[B71] Isaac J. T. Nicoll R. A. Malenka R. C. (1995). Evidence for silent synapses: implications for the expression of LTP. Neuron 15, 427–434. 10.1016/0896-6273(95)90046-2 7646894

[B72] Jacobs S. E. Berg M. Hunt R. Tarnow-Mordi W. O. Inder T. E. Davis P. G. (2013). Cooling for newborns with hypoxic ischaemic encephalopathy. Cochrane Database Syst. Rev. 1:CD003311. 10.1002/14651858.CD003311.pub3 14583966

[B73] Ji X. Y. Zingg B. Mesik L. Xiao Z. Zhang L. I. Tao H. W. (2016). Thalamocortical innervation pattern in mouse auditory and visual cortex: laminar and cell-type specificity. Cereb. Cortex 26, 2612–2625. 10.1093/cercor/bhv099 25979090 PMC4869808

[B74] Judaš M. Sedmak G. Pletikos M. Jovanov-Milošević N. (2010). Populations of subplate and interstitial neurons in fetal and adult human telencephalon. J. Anat. 217, 381–399. 10.1111/j.1469-7580.2010.01284.x 20979586 PMC2992415

[B75] Kanold P. O. (2009). Subplate neurons: crucial regulators of cortical development and plasticity. Front. Neuroanat. 3:16. 10.3389/neuro.05.016.2009 19738926 PMC2737439

[B76] Kanold P. O. (2019). The first cortical circuits: subplate neurons lead the way and shape cortical organization. Neuroforum 25, 15–23. 10.1515/nf-2018-0010

[B77] Kanold P. O. Kara P. Reid R. C. Shatz C. J. (2003). Role of subplate neurons in functional maturation of visual cortical columns. Science 301, 521–525. 10.1126/science.1084152 12881571

[B78] Kanold P. O. Luhmann H. J. (2010). The subplate and early cortical circuits. Annu. Rev. Neurosci. 33, 23–48. 10.1146/annurev-neuro-060909-153244 20201645

[B79] Kanold P. O. Shatz C. J. (2006). Subplate neurons regulate maturation of cortical inhibition and outcome of ocular dominance plasticity. Neuron 51, 627–638. 10.1016/j.neuron.2006.07.008 16950160

[B80] Kerchner G. A. Nicoll R. A. (2008). Silent synapses and the emergence of a postsynaptic mechanism for LTP. Nat. Rev. Neurosci. 9, 813–825. 10.1038/nrn2501 18854855 PMC2819160

[B81] Khazipov R. Luhmann H. J. (2006). Early patterns of electrical activity in the developing cerebral cortex of humans and rodents. Trends Neurosci. 29, 414–418. 10.1016/j.tins.2006.05.007 16713634

[B82] Khazipov R. Minlebaev M. Valeeva G. (2013). Early γ oscillations. Neuroscience 250, 240–252. 10.1016/j.neuroscience.2013.07.019 23872391

[B83] Khazipov R. Sirota A. Leinekugel X. Holmes G. L. Ben-Ari Y. Buzsáki G. (2004). Early motor activity drives spindle bursts in the developing somatosensory cortex. Nature 432, 758–761. 10.1038/nature03132 15592414

[B84] Kilb W. Kirischuk S. Luhmann H. J. (2011). Electrical activity patterns and the functional maturation of the neocortex. Eur. J. Neurosci. 34, 1677–1686. 10.1111/j.1460-9568.2011.07878.x 22103424

[B85] Kotak V. C. Sadahiro M. Fall C. P. (2007). Developmental expression of endogenous oscillations and waves in the auditory cortex involves calcium, gap junctions, and GABA. Neuroscience 146, 1629–1639. 10.1016/j.neuroscience.2007.03.039 17478052

[B86] Krmpotić-Nemanić J. Kostović I. Kelović Z. Nemanić D. Mrzljak L. (1983). Development of the human fetal auditory cortex: growth of afferent fibres. Acta Anat. 116, 69–73. 10.1159/000145727 6858605

[B87] Kullmann D. M. (2003). Silent synapses: what are they telling us about long-term potentiation? Philos. Trans. R. Soc. Lond. B Biol. Sci. 358, 727–733. 10.1098/rstb.2002.1229 12740119 PMC1693148

[B88] Kullmann D. M. Asztely F. (1998). Extrasynaptic glutamate spillover in the hippocampus: evidence and implications. Trends Neurosci. 21, 8–14. 10.1016/s0166-2236(97)01150-8 9464678

[B89] Laviola G. Adriani W. Rea M. Aloe L. Alleva E. (2004). Social withdrawal, neophobia, and stereotyped behavior in developing rats exposed to neonatal asphyxia. Psychopharmacology 175, 196–205. 10.1007/s00213-004-1800-3 14985924

[B92] Liao D. Hessler N. A. Malinow R. (1995). Activation of postsynaptically silent synapses during pairing-induced LTP in CA1 region of hippocampal slice. Nature 375, 400–404. 10.1038/375400a0 7760933

[B90] Liao C. C. Lee L. J. (2011). Neonatal fluoxetine exposure affects the action potential properties and dendritic development in cortical subplate neurons of rats. Toxicol. Lett. 207, 314–321. 10.1016/j.toxlet.2011.09.028 21986067

[B91] Liao C. C. Lee L. J. (2014). Presynaptic 5-HT1B receptor-mediated synaptic suppression to the subplate neurons in the somatosensory cortex of neonatal rats. Neuropharmacology 77, 81–89. 10.1016/j.neuropharm.2013.08.040 24055501

[B93] Lu W. Constantine-Paton M. (2004). Eye opening rapidly induces synaptic potentiation and refinement. Neuron 43, 237–249. 10.1016/j.neuron.2004.06.031 15260959

[B94] Luhmann H. J. Khazipov R. (2018). Neuronal activity patterns in the developing barrel cortex. Neuroscience 368, 256–267. 10.1016/j.neuroscience.2017.05.025 28528963

[B95] Luhmann H. J. Kirischuk S. Kilb W. (2018). The superior function of the subplate in early neocortical development. Front. Neuroanat. 12:97. 10.3389/fnana.2018.00097 30487739 PMC6246655

[B96] Luhmann H. J. Kral T. Heinemann U. (1993). Influence of hypoxia on excitation and GABAergic inhibition in mature and developing rat neocortex. Exp. Brain Res. 97, 209–224. 10.1007/bf00228690 7908647

[B97] Luhmann H. J. Sinning A. Yang J. W. Reyes-Puerta V. Stüttgen M. C. Kirischuk S.. (2016). Spontaneous neuronal activity in developing neocortical networks: from single cells to large-scale interactions. Front. Neural Circuits 10:40. 10.3389/fncir.2016.00040 27252626 PMC4877528

[B98] Ma Y. Hioki H. Konno M. Pan S. Nakamura H. Nakamura K. C.. (2011). Expression of gap junction protein connexin36 in multiple subtypes of GABAergic neurons in adult rat somatosensory cortex. Cereb. Cortex 21, 2639–2649. 10.1093/cercor/bhr051 21467210

[B99] Mabunga D. F. Gonzales E. L. Kim J. W. Kim K. C. Shin C. Y. (2015). Exploring the validity of valproic acid animal model of autism. Exp. Neurobiol. 24, 285–300. 10.5607/en.2015.24.4.285 26713077 PMC4688329

[B100] Marcano-Reik A. J. Blumberg M. S. (2008). The corpus callosum modulates spindle-burst activity within homotopic regions of somatosensory cortex in newborn rats. Eur. J. Neurosci. 28, 1457–1466. 10.1111/j.1460-9568.2008.06461.x 18973571 PMC2669778

[B101] Marx M. Qi G. Hanganu-Opatz I. L. Kilb W. Luhmann H. J. Feldmeyer D. (2017). Neocortical layer 6B as a remnant of the subplate—a morphological comparison. Cereb. Cortex 27, 1011–1026. 10.1093/cercor/bhv279 26637449

[B102] McConnell S. K. Ghosh A. Shatz C. J. (1994). Subplate pioneers and the formation of descending connections from cerebral cortex. J. Neurosci. 14, 1892–1907. 10.1523/JNEUROSCI.14-04-01892.1994 7512631 PMC6577121

[B103] McFadden K. Minshew N. J. (2013). Evidence for dysregulation of axonal growth and guidance in the etiology of ASD. Front. Hum. Neurosci. 7:671. 10.3389/fnhum.2013.00671 24155705 PMC3804918

[B104] McQuillen P. S. Sheldon R. A. Shatz C. J. Ferriero D. M. (2003). Selective vulnerability of subplate neurons after early neonatal hypoxia-ischemia. J. Neurosci. 23, 3308–3315. 10.1523/JNEUROSCI.23-08-03308.2003 12716938 PMC6742293

[B105] Mechawar N. Descarries L. (2001). The cholinergic innervation develops early and rapidly in the rat cerebral cortex: a quantitative immunocytochemical study. Neuroscience 108, 555–567. 10.1016/s0306-4522(01)00389-x 11738494

[B106] Meinecke D. L. Rakic P. (1992). Expression of GABA and GABA_A_ receptors by neurons of the subplate zone in developing primate occipital cortex: evidence for transient local circuits. J. Comp. Neurol. 317, 91–101. 10.1002/cne.903170107 1315345

[B107] Meister M. Wong R. O. Baylor D. A. Shatz C. J. (1991). Synchronous bursts of action potentials in ganglion cells of the developing mammalian retina. Science 252, 939–943. 10.1126/science.2035024 2035024

[B108] Meng X. Kao J. P. Kanold P. O. (2014). Differential signaling to subplate neurons by spatially specific silent synapses in developing auditory cortex. J. Neurosci. 34, 8855–8864. 10.1523/jneurosci.0233-14.2014 24966385 PMC4069358

[B109] Meyer H. S. Wimmer V. C. Hemberger M. Bruno R. M. de Kock C. P. Frick A.. (2010). Cell type-specific thalamic innervation in a column of rat vibrissal cortex. Cereb. Cortex 20, 2287–2303. 10.1093/cercor/bhq069 20534783 PMC2936808

[B110] Mikhailova A. Sunkara N. McQuillen P. S. (2017). Unbiased quantification of subplate neuron loss following neonatal hypoxia-ischemia in a rat model. Dev. Neurosci. 39, 171–181. 10.1159/000460815 28434006 PMC5519415

[B111] Minlebaev M. Ben-Ari Y. Khazipov R. (2009). NMDA receptors pattern early activity in the developing barrel cortex *in vivo*. Cereb. Cortex 19, 688–696. 10.1093/cercor/bhn115 18663251

[B112] Minlebaev M. Colonnese M. Tsintsadze T. Sirota A. Khazipov R. (2011). Early γ oscillations synchronize developing thalamus and cortex. Science 334, 226–229. 10.1126/science.1210574 21998388

[B113] Molnár Z. Kurotani T. Higashi S. Yamamoto N. Toyama K. (2003). Development of functional thalamocortical synapses studied with current source-density analysis in whole forebrain slices in the rat. Brain Res. Bull. 60, 355–371. 10.1016/s0361-9230(03)00061-3 12781324

[B114] Mrzljak L. Uylings H. B. Kostovic I. Van Eden C. G. (1988). Prenatal development of neurons in the human prefrontal cortex: I. A qualitative Golgi study. J. Comp. Neurol. 271, 355–386. 10.1002/cne.902710306 2454966

[B115] Murata Y. Colonnese M. T. (2016). An excitatory cortical feedback loop gates retinal wave transmission in rodent thalamus. Elife 5:e18816. 10.7554/elife.18816 27725086 PMC5059135

[B116] Myakhar O. Unichenko P. Kirischuk S. (2011). GABAergic projections from the subplate to Cajal-Retzius cells in the neocortex. Neuroreport 22, 525–529. 10.1097/wnr.0b013e32834888a4 21666518

[B117] Nadebaum C. Anderson V. A. Vajda F. Reutens D. C. Barton S. Wood A. G. (2011). Language skills of school-aged children prenatally exposed to antiepileptic drugs. Neurology 76, 719–726. 10.1212/wnl.0b013e31820d62c7 21339499

[B118] Nagode D. A. Meng X. Winkowski D. E. Smith E. Khan-Tareen H. Kareddy V.. (2017). Abnormal development of the earliest cortical circuits in a mouse model of autism spectrum disorder. Cell Rep. 18, 1100–1108. 10.1016/j.celrep.2017.01.006 28147267 PMC5488290

[B119] Nakazawa M. Koh T. Kani K. Maeda T. (1992). Transient patterns of serotonergic innervation in the rat visual cortex: normal development and effects of neonatal enucleation. Dev. Brain Res. 66, 77–90. 10.1016/0165-3806(92)90143-k 1600633

[B120] Nicolini C. Fahnestock M. (2018). The valproic acid-induced rodent model of autism. Exp. Neurol. 299, 217–227. 10.1016/j.expneurol.2017.04.017 28472621

[B121] Niculescu D. Lohmann C. (2014). Gap junctions in developing thalamic and neocortical neuronal networks. Cereb. Cortex 24, 3097–3106. 10.1093/cercor/bht175 23843439 PMC4224240

[B122] Nowak L. Bregestovski P. Ascher P. Herbet A. Prochiantz A. (1984). Magnesium gates glutamate-activated channels in mouse central neurones. Nature 307, 462–465. 10.1038/307462a0 6320006

[B123] O’Leary D. D. Borngasser D. (2006). Cortical ventricular zone progenitors and their progeny maintain spatial relationships and radial patterning during preplate development indicating an early protomap. Cereb. Cortex 16, i46–i56. 10.1093/cercor/bhk019 16766707

[B124] O’Leary D. D. Chou S. J. Hamasaki T. Sahara S. Takeuchi A. Thuret S.. (2007). Regulation of laminar and area patterning of mammalian neocortex and behavioural implications. Novartis Found. Symp. 288, 141–159; discussion 159–164, 276–181. 10.1002/9780470994030.ch11 18494257

[B125] O’Reilly J. P. Haddad G. G. (1996). Chronic hypoxia *in vivo* renders neocortical neurons more vulnerable to subsequent acute hypoxic stress. Brain Res. 711, 203–210. 10.1016/0006-8993(95)01396-2 8680864

[B126] Pedraza M. Hoerder-Suabedissen A. Albert-Maestro M. A. Molnar Z. De Carlos J. A. (2014). Extracortical origin of some murine subplate cell populations. Proc. Natl. Acad. Sci. U S A 111, 8613–8618. 10.1073/pnas.1323816111 24778253 PMC4060693

[B127] Peinado A. Yuste R. Katz L. C. (1993a). Gap junctional communication and the development of local circuits in neocortex. Cereb. Cortex 3, 488–498. 10.1093/cercor/3.5.488 8260815

[B128] Peinado A. Yuste R. Katz L. C. (1993b). Extensive dye coupling between rat neocortical neurons during the period of circuit formation. Neuron 10, 103–114. 10.1016/0896-6273(93)90246-n 8427699

[B129] Penn A. A. Riquelme P. A. Feller M. B. Shatz C. J. (1998). Competition in retinogeniculate patterning driven by spontaneous activity. Science 279, 2108–2112. 10.1126/science.279.5359.2108 9516112

[B130] Piñon M. C. Jethwa A. Jacobs E. Campagnoni A. Molnar Z. (2009). Dynamic integration of subplate neurons into the cortical barrel field circuitry during postnatal development in the Golli-tau-eGFP (GTE) mouse. J. Physiol. 587, 1903–1915. 10.1113/jphysiol.2008.167767 19289548 PMC2689332

[B131] Pisani A. Calabresi P. Bernardi G. (1997). Hypoxia in striatal and cortical neurones: membrane potential and Ca^2+^ measurements. Neuroreport 8, 1143–1147. 10.1097/00001756-199703240-00017 9175102

[B132] Postma F. Liu C. H. Dietsche C. Khan M. Lee H. K. Paul D.. (2011). Electrical synapses formed by connexin36 regulate inhibition- and experience-dependent plasticity. Proc. Natl. Acad. Sci. U S A 108, 13770–13775. 10.1073/pnas.1100166108 21804029 PMC3158176

[B133] Ranasinghe S. Or G. Wang E. Y. Ievins A. McLean M. A. Niell C. M.. (2015). Reduced cortical activity impairs development and plasticity after neonatal hypoxia ischemia. J. Neurosci. 35, 11946–11959. 10.1523/jneurosci.2682-14.2015 26311776 PMC4549405

[B134] Reep R. L. (2000). Cortical layer VII and persistent subplate cells in mammalian brains. Brain Behav. Evol. 56, 212–234. 10.1159/000047206 11155000

[B135] Rennie J. M. Hagmann C. F. Robertson N. J. (2007). Outcome after intrapartum hypoxic ischaemia at term. Semin. Fetal Neonatal Med. 12, 398–407. 10.1016/j.siny.2007.07.006 17825633

[B136] Roerig B. Feller M. B. (2000). Neurotransmitters and gap junctions in developing neural circuits. Brain Res. Rev. 32, 86–114. 10.1016/s0165-0173(99)00069-7 10751659

[B137] Rogers C. E. Lean R. E. Wheelock M. D. Smyser C. D. (2018). Aberrant structural and functional connectivity and neurodevelopmental impairment in preterm children. J. Neurodev. Disord. 10:38. 10.1186/s11689-018-9253-x 30541449 PMC6291944

[B138] Rosen A. S. Morris M. E. (1994). Influence of temperature on anoxic responses of neocortical pyramidal neurons. Neuroscience 63, 949–955. 10.1016/0306-4522(94)90563-0 7700518

[B139] Rumpel S. Hatt H. Gottmann K. (1998). Silent synapses in the developing rat visual cortex: evidence for postsynaptic expression of synaptic plasticity. J. Neurosci. 18, 8863–8874. 10.1523/JNEUROSCI.18-21-08863.1998 9786992 PMC6793542

[B140] Rumpel S. Kattenstroth G. Gottmann K. (2004). Silent synapses in the immature visual cortex: layer-specific developmental regulation. J. Neurophysiol. 91, 1097–1101. 10.1152/jn.00443.2003 14762153

[B141] Ruthazer E. S. Akerman C. J. Cline H. T. (2003). Control of axon branch dynamics by correlated activity *in vivo*. Science 301, 66–70. 10.1126/science.1082545 12843386

[B142] Sethuramanujam S. Yao X. deRosenroll G. Briggman K. L. Field G. D. Awatramani G. B. (2017). “Silent” NMDA synapses enhance motion sensitivity in a mature retinal circuit. Neuron 96, 1099.e3–1111.e3. 10.1016/j.neuron.2017.09.058 29107522 PMC5975974

[B143] Shah R. D. Crair M. C. (2008). Retinocollicular synapse maturation and plasticity are regulated by correlated retinal waves. J. Neurosci. 28, 292–303. 10.1523/jneurosci.4276-07.2008 18171946 PMC6671137

[B144] Sheikh A. Meng X. Liu J. Mikhailova A. Kao J. P. Y. McQuillen P. S.. (2019). Neonatal hypoxia-ischemia causes functional circuit changes in subplate neurons. Cereb. Cortex 29, 765–776. 10.1093/cercor/bhx358 29365081 PMC6676960

[B145] Sherman S. M. (2012). Thalamocortical interactions. Curr. Opin. Neurobiol. 22, 575–579. 10.1016/j.conb.2012.03.005 22498715 PMC3398163

[B146] Sherman S. M. (2017). Functioning of circuits connecting thalamus and cortex. Compr. Physiol. 7, 713–739. 10.1002/cphy.c160032 28333385

[B147] Siegel F. Heimel J. A. Peters J. Lohmann C. (2012). Peripheral and central inputs shape network dynamics in the developing visual cortex *in vivo*. Curr. Biol. 22, 253–258. 10.1016/j.cub.2011.12.026 22264606

[B148] Stewart C. V. Plenz D. (2008). Homeostasis of neuronal avalanches during postnatal cortex development *in vitro*. J. Neurosci. Methods 169, 405–416. 10.1016/j.jneumeth.2007.10.021 18082894 PMC2743406

[B149] Stoner R. Chow M. L. Boyle M. P. Sunkin S. M. Mouton P. R. Roy S.. (2014). Patches of disorganization in the neocortex of children with autism. N. Engl. J. Med. 370, 1209–1219. 10.1056/NEJMoa1307491 24670167 PMC4499461

[B150] Suárez-Solá M. L. González-Delgado F. J. Pueyo-Morlans M. Medina-Bolívar O. C. Hernández-Acosta N. C. González-Gómez M.. (2009). Neurons in the white matter of the adult human neocortex. Front. Neuroanat. 3:7. 10.3389/neuro.05.007.2009 19543540 PMC2697018

[B152] Sun J. J. Kilb W. Luhmann H. J. (2010). Self-organization of repetitive spike patterns in developing neuronal networks *in vitro*. Eur. J. Neurosci. 32, 1289–1299. 10.1111/j.1460-9568.2010.07383.x 20846326

[B153] Sun J. J. Luhmann H. J. (2007). Spatio-temporal dynamics of oscillatory network activity in the neonatal mouse cerebral cortex. Eur. J. Neurosci. 26, 1995–2004. 10.1111/j.1460-9568.2007.05819.x 17868367

[B151] Sun H. Takesian A. E. Wang T. T. Lippman-Bell J. J. Hensch T. K. Jensen F. E. (2018). Early seizures prematurely unsilence auditory synapses to disrupt thalamocortical critical period plasticity. Cell Rep. 23, 2533–2540. 10.1016/j.celrep.2018.04.108 29847785 PMC6446922

[B154] Sun W. Tan Z. Mensh B. D. Ji N. (2016). Thalamus provides layer 4 of primary visual cortex with orientation- and direction-tuned inputs. Nat. Neurosci. 19, 308–315. 10.1038/nn.4196 26691829 PMC4731241

[B155] Supèr H. Soriano E. Uylings H. B. (1998). The functions of the preplate in development and evolution of the neocortex and hippocampus. Brain Res. Rev. 27, 40–64. 10.1016/s0165-0173(98)00005-8 9639671

[B156] Tolner E. A. Sheikh A. Yukin A. Y. Kaila K. Kanold P. O. (2012). Subplate neurons promote spindle bursts and thalamocortical patterning in the neonatal rat somatosensory cortex. J. Neurosci. 32, 692–702. 10.1523/jneurosci.1538-11.2012 22238105 PMC3517992

[B157] Tolonen M. Palva J. M. Andersson S. Vanhatalo S. (2007). Development of the spontaneous activity transients and ongoing cortical activity in human preterm babies. Neuroscience 145, 997–1006. 10.1016/j.neuroscience.2006.12.070 17307296

[B158] Torres-Reveron J. Friedlander M. J. (2007). Properties of persistent postnatal cortical subplate neurons. J. Neurosci. 27, 9962–9974. 10.1523/jneurosci.1536-07.2007 17855610 PMC6672634

[B159] Tritsch N. X. Yi E. Gale J. E. Glowatzki E. Bergles D. E. (2007). The origin of spontaneous activity in the developing auditory system. Nature 450, 50–55. 10.1038/nature06233 17972875

[B160] Vanhatalo S. Kaila K. (2006). Development of neonatal EEG activity: from phenomenology to physiology. Semin. Fetal Neonatal Med. 11, 471–478. 10.1016/j.siny.2006.07.008 17018268

[B161] Vanhatalo S. Tallgren P. Andersson S. Sainio K. Voipio J. Kaila K. (2002). DC-EEG discloses prominent, very slow activity patterns during sleep in preterm infants. Clin. Neurophysiol. 113, 1822–1825. 10.1016/s1388-2457(02)00292-4 12417237

[B162] Venance L. Rozov A. Blatow M. Burnashev N. Feldmeyer D. Monyer H. (2000). Connexin expression in electrically coupled postnatal rat brain neurons. Proc. Natl. Acad. Sci. U S A 97, 10260–10265. 10.1073/pnas.160037097 10944183 PMC27858

[B163] Viswanathan S. Bandyopadhyay S. Kao J. P. Kanold P. O. (2012). Changing microcircuits in the subplate of the developing cortex. J. Neurosci. 32, 1589–1601. 10.1523/jneurosci.4748-11.2012 22302801 PMC3517995

[B164] Viswanathan S. Sheikh A. Looger L. L. Kanold P. O. (2017). Molecularly defined subplate neurons project both to thalamocortical recipient layers and thalamus. Cereb. Cortex 27, 4759–4768. 10.1093/cercor/bhw271 27655928 PMC6059176

[B165] Voigt T. de Lima A. D. (1991). Serotoninergic innervation of the ferret cerebral cortex. J. Comp. Neurol. 314, 415–428. 10.1002/cne.903140215 1787183

[B167] Wang H. C. Bergles D. E. (2015). Spontaneous activity in the developing auditory system. Cell Tissue Res. 361, 65–75. 10.1007/s00441-014-2007-5 25296716 PMC7046314

[B166] Wang D. D. Kriegstein A. R. (2008). GABA regulates excitatory synapse formation in the neocortex via NMDA receptor activation. J. Neurosci. 28, 5547–5558. 10.1523/jneurosci.5599-07.2008 18495889 PMC2684685

[B168] Wess J. M. Isaiah A. Watkins P. V. Kanold P. O. (2017). Subplate neurons are the first cortical neurons to respond to sensory stimuli. Proc. Natl. Acad. Sci. U S A 114, 12602–12607. 10.1073/pnas.1710793114 29114043 PMC5703299

[B169] Winnubst J. Cheyne J. E. Niculescu D. Lohmann C. (2015). Spontaneous activity drives local synaptic plasticity *in vivo*. Neuron 87, 399–410. 10.1016/j.neuron.2015.06.029 26182421

[B170] Yang J. W. An S. Sun J. J. Reyes-Puerta V. Kindler J. Berger T.. (2013). Thalamic network oscillations synchronize ontogenetic columns in the newborn rat barrel cortex. Cereb. Cortex 23, 1299–1316. 10.1093/cercor/bhs103 22593243

[B171] Yang J. W. Hanganu-Opatz I. L. Sun J. J. Luhmann H. J. (2009). Three patterns of oscillatory activity differentially synchronize developing neocortical networks *in vivo*. J. Neurosci. 29, 9011–9025. 10.1523/jneurosci.5646-08.2009 19605639 PMC6665441

[B172] Yang J. W. Reyes-Puerta V. Kilb W. Luhmann H. J. (2016). Spindle bursts in neonatal rat cerebral cortex. Neural Plast. 2016:3467832. 10.1155/2016/3467832 27034844 PMC4806652

[B173] Yao X. H. Wang M. He X. N. He F. Zhang S. Q. Lu W.. (2016). Electrical coupling regulates layer 1 interneuron microcircuit formation in the neocortex. Nat. Commun. 7:12229. 10.1038/ncomms12229 27510304 PMC4987578

[B174] Zhao C. Kao J. P. Kanold P. O. (2009). Functional excitatory microcircuits in neonatal cortex connect thalamus and layer 4. J. Neurosci. 29, 15479–15488. 10.1523/jneurosci.4471-09.2009 20007472 PMC3539415

[B175] Zhou C. Lippman J. J. Sun H. Jensen F. E. (2011). Hypoxia-induced neonatal seizures diminish silent synapses and long-term potentiation in hippocampal CA1 neurons. J. Neurosci. 31, 18211–18222. 10.1523/jneurosci.4838-11.2011 22171027 PMC3282023

